# Traumatic Neurasthenia and Mental Disease

**Published:** 1915-03-13

**Authors:** 


### TRAUMATIC NEURASTHENIA AND MENTAL DISEASE.

### The Need for more Precise Definition.

The exact definition of the term "neurasthenia" is still a matter of dispute, and it is, therefore, with some caution that a particular variety of this condition is here discussed. If it be granted, however, that, like many other expressions made use of in medicine and in other sciences, the name is used as a convenient basis for discussion until more confident knowledge is possible, no objection can be taken to its employment. This, at all events, is indubitable, that in certain cases traumatisms of various kinds have brought about disturbances in the activities of the nervous system, and that these disturbances may be conveniently included under the term "neurasthenia."

In many of these cases the responsibility is to be traced to a railway or other collision, and in some instances the amount of violence has been slight; just now, the condition is being noted in a proportion of those exposed to the strain and concussion of the war on the battlefield. It is a curious and interesting fact that where some obvious injury, such as a fractured bone, has resulted from violence, neurasthenia often, or even usually, does not supervene. It may be that in such instances the stimulus of hope of recovery from the visible morbid process counteracts the influences which would otherwise disturb the nervous functions. Where the trauma is more marked, the changes in the nerve-cells are so profound as to prevent this factor from operating until a later stage. The element of hope—or of hope deferred—is no doubt a factor in some cases where litigation in the shape of a claim for damages is at issue. In the less pronounced exhibitions of the neurasthenic symptoms extremely difficult problems are presented to the investigator; the possibility of malingering has to be taken into account, and much injustice may be done if such cases are not subjected to the most minute and scrupulous examination. Where the symptoms are more marked, however, there is no difficulty in deciding that some grave interference with nerve-function has resulted from the injury which has been sustained. Occasionally this disorder of function is so pronounced as to bring about a definite traumatic neurosis, necessitating the certification of the sufferer as a person of unsound mind.

When one thinks of the extreme delicacy of the nervous mechanism the amazing fact is that mental disorder so seldom follows after even grave injuries to the brain. Clouston states that in nine years the cases of traumatic insanity in the Royal Edinburgh Mental Hospital formed only one-third per cent. of the admissions: and other observers are led to a parallel conclusion. If one considers the large numbers of accidents of all kinds which occur without any subsequent nervous disturbance, it is obvious that some factor other than the actual trauma enters into the causation of this neurosis; just as, while syphilis is common, general paralysis of the insane, which is one of its results, is relatively rare. There must, in other words, be some predisposition, resulting perhaps from an inherited nervous instability.

It may be asserted that those persons who develop such marked mental symptoms as to necessitate certification are not really neurasthenics. If it is desired that the term "neurasthenia" should be restricted to less obvious derangements, the assertion is justified: and the expression "traumatic neurosis" may be used to designate these graver cases. But the conditions shade indefinably from one into the other; and similar accidents which may produce in one case a transient concussion will in another result in neurasthenia of the accepted form, or in a third will give rise to a definite neurosis or to epileptic insanity. Indeed, such conditions as those just named may succeed one another in the same case, and the symptoms of concussion when they pass off may be followed by some lasting mental derangement. The sequence may be comparatively rapid; on the other hand, the intellectual disorder may not develop until some weeks or months later.

### Effects of Shell-fire.

There is usually depression as a prominent symptom, and with this there may be hypochondriacal ideas, vague pains and aches, or definite assertions of disease or injury, the presence of which, however, careful examination fails to disclose. There is a feeling of inability to carry on the accustomed work or adequately to fulfil the usual duties, and as a result of this there may be suggestions on the part of the patient of suicide or even actual attempts at self-destruction. In other cases there is marked confusion; inability to converse coherently; automatism—as in a case reported from the seat of battle of a soldier who sat ceaselessly beating the bottom of a bucket for hours, apparently oblivious of everything that was taking place around him; vague fears and obsessions. In others again the nervous symptoms may show themselves in intense excitement, restlessness, and a general condition of acute mania, with hallucinations of sight and hearing. It appears from this as if the whole gamut of morbid mental symptoms may be gone through by the various patients; and this is indeed so, as the records of the effects, in particular, of the unendurable din of bursting shells go to prove. "The hardest task of the soldier," says one correspondent, "is to get used to the appalling noise of the artillery": the comment is made in regard to the arrival of two goods waggons "full of German soldiers who have gone mad owing to the intolerable noise of the battlefield." Another writes: "So terrific have our shells been that over a score of the 5,000 prisoners . . . have been almost insane. . . . They were paralysed with fright." Of the Poles during the German invasion we read that "Many have died or have become crazed through fright": this was among the civil population.

The course which any particular case will follow is more dependent upon idiosyncrasy than upon the exciting cause, and the same holds true in regard to prognosis. The whole subject is too obscure to permit of dogmatism. It is obvious that in the early stages much caution must be exercised, as it is practically impossible to state accurately to what extent the involvement of the nervous system may proceed; and even when the neurasthenia or the neurosis has apparently fully developed it is still difficult to prophesy how long a time will elapse before recovery takes place. Some cases recover in the course of a few months; in others recovery more apparent than real takes place—undue muscular excitability with a marked tendency to rapid fatigue remaining as a persistent symptom. Associated with this there is inability to concentrate the attention, so that the individual is unable again to pursue his usual avocations.

In the matter of treatment this has to be, in the accepted phrase, on general lines. Rest, even to the extent at first of keeping the patient in bed, carefully regulated diet, and anything that tends to improve the general health; absence of worry, and towards this end the patient should be protected from those irritating relatives and friends who insist on "rousing him," or who maintain that his lassitude is laziness; and the other methods may be resorted to whereby nutrition is improved and the patient is interested and occupied without fatigue being engendered. In some cases treatment by suggestion has proved of value. The outlook is hopeful, and the patient should be told so; on the other hand, patience must be exercised by him and by those in attendance on him. A return to employment should be delayed for some little time after apparent recovery and until a moderate amount of work has been attempted in a tentative way without recurrence of the neurasthenic symptoms.

It is frankly admitted that this attempt to deal with the subject is somewhat vague and inconclusive. But, just as rigid classification is impossible, on account of the difficulty of accurately delimiting the particular symptom-complex, so prognosis and treatment must necessarily be inchoate and tentative until further investigation and experiment make the way clear. One of the ways in which we hope medical science may gain from the present war is in the clinical material thus suddenly placed at its disposal for the investigation of such conditions as these.

